# Use of the Pediatric Symptom Checklist for the detection of psychosocial problems in preventive child healthcare

**DOI:** 10.1186/1471-2458-6-197

**Published:** 2006-07-27

**Authors:** Sijmen A Reijneveld, Anton GC Vogels, Femke Hoekstra, Matty R Crone

**Affiliations:** 1 University Medical Center Groningen, University of Groningen, Department of Health Sciences, P.O. Box 196, 9700 AD Groningen, The Netherlands; 2 TNO (Netherlands Organisation of Applied Scientific Research) Quality of Life, Department of Child Health, Leiden, The Netherlands; 3 Amsterdam Municipal Health Service, Amsterdam, The Netherlands

## Abstract

**Background:**

Early detection and treatment of psychosocial problems by preventive child healthcare may lead to considerable health benefits, and a short questionnaire could support this aim. The aim of this study was to assess whether the Dutch version of the US Pediatric Symptom checklist (PSC) is valid and suitable for the early detection of psychosocial problems among children.

**Methods:**

We included 687 children (response 84.3%) aged 7–12 undergoing routine health assessments in nine Preventive Child Health Services across the Netherlands. Child health professionals interviewed and examined children and parents. Before the interview, parents completed an authorised Dutch translation of the PSC and the Child Behavior Checklist (CBCL). The CBCL and data on the child's current treatment status were used as criteria for the validity of the PSC.

**Results:**

The consistency of the Dutch PSC was good (Cronbach alpha 0.89). The area under the ROC curve using the CBCL as a criterion was 0.94 (95% confidence interval 0.92 to 0.96). At the US cut-off (28 and above), the prevalence rate of an increased score and sensitivity were lower than in the USA. At a lower cut-off (22 and above), sensitivity and specificity were similar to that of the US version (71.7% and 93.0% respectively). Information on the PSC also helped in the identification of children with elevated CBCL Total Problems Scores, above solely clinical judgment.

**Conclusion:**

The PSC is also useful for the early detection of psychosocial problems in preventive child healthcare outside the USA, especially with an adjusted cut-off.

## Background

Early detection and treatment of psychosocial problems may lead to considerable health benefits. Psychosocial problems have a high prevalence rate and lead to high costs of disease [1]. They also cause substantial restrictions in daily functioning in later life and are the major cause of long-term work disability in young adults [1]. Only a minority of children with psychological or psychosocial problems are under treatment [2-4]. If untreated, problems are likely to persist in later life and can lead to serious limitations in daily functioning [2,5]. Research has shown that early detection and treatment improves these children's prognosis substantially [6,7], but a complete analysis of its cost effectiveness has yet to be carried out.

The community child health service is an ideal setting for the early detection of psychosocial problems among children as routine health examinations are provided through it for the entire population, as a standardised part of preventive child healthcare (PCH). In the Netherlands, municipalities are obliged by law to guarantee proper access to this type of care, free of charge.

However, the predictive value of early detection of psychosocial problems by PCH is still too low [3,4]. For instance, Brugman et al. show that even though Dutch PCH identifies psychosocial problems in 25% of all children of school age, they miss 43% of the children with a clinical score on the Child Behavior Checklist (CBCL) [3]. Similarly, Murphy et al. reported that paediatricians had identified psychosocial problems in less than half of the children with elevated scores on the Pediatric Symptom Checklist (PSC) or the Child Global Assessment Scale [8].

The PSC is a 35-item parent-completed questionnaire that supports the identification of psychosocial problems by paediatricians [8-14]. It takes less than 5 minutes to complete and score, and reflects the parent's impression of his or her child's psychosocial functioning. Its validity has been demonstrated in various paediatric settings in the USA, nationally [10] in inner-city children [8], in Hispanic children [11,12] and in children of substance-abusing parents [14]. Moreover, the PSC has recently been used as an outcome measure in the assessment of interventions to reduce the impact of trauma [13]. Given its good validity and applicability in US community child health services, the PSC is a likely candidate for use in other countries with similar systems of preventive child healthcare, such as the Netherlands.

The aim of this study was to assess the test properties of the Dutch version of the PSC and determine whether it would be suitable for and contribute to the early detection of psychosocial problems in children aged 7–12 by PCH.

## Methods

This study is based on a community sample of children for whom PSC and CBCL data are available, and data on the identification and management of psychosocial problems by CHPs.

### Population

The sample was obtained using a two-stage selection procedure. In the first stage, a national sample from 9 of the 41 Dutch Preventive Child Health Services was taken. In the second stage, each Service provided a sample of children aged 7–12 who were invited for routine well-child examinations. We aimed at a sample size of 700 respondents for evaluation, as earlier studies [15,16] demonstrated that short questionnaires used in PCH settings allow for an area under the ROC curves (AUC) of about 0.90 with a clinical CBCL Total Problems score as criterion. A sample of 700 suffices to estimate this AUC with a 95% confidence interval of +/- 0.02.

Of the total sample of 815 eligible children, 687 participated and 674 provided complete data on both questionnaires (84.3% and 82.7% of the original sample, respectively). Both groups were representative of the total sample regarding age and gender, but non-response was higher for children of immigrant/minority origin (27.4% vs. 12.2%). Analyses were restricted to children with complete data for both questionnaires to make interpretation easier.

### Data collection

The data were collected according to a standardised procedure during routine well-child examinations, from September 2004 to July 2005. The study was performed in compliance with the Helsinki Declaration [17]. The design of the study was approved by the local TNO Medical-Ethical Committee and includes verbal informed consent by parents.

The PSC [10] and the CBCL [18,19] were mailed to children, along with the standard invitation for the preventive health assessment. Before attending the assessments, parents completed the questionnaires, placed them in sealed envelopes and gave them to the CHPs, who in turn passed them on to the researchers without opening them (in contrast with routine use, where the CHP would partially base the interview on the information from the PSC). The CHP interviewed each child and its parents regarding mental health and background, and examined each child. After each assessment, the CHP answered the following question: 'Does the child have a psychosocial problem, at this moment?' (yes, no) and scored its severity (mild, moderate or severe) and the type of problems identified using a pre-coded list. Children who only had risk indicators for the development of psychosocial problems, such as having parents with psychiatric problems or other family problems, had to be coded as having no problems.

The PSC was translated following the procedure proposed by Guillemin et al. [20]. Firstly, the original US English version of the questionnaire was translated into Dutch by three certified translators working independently of each other. Secondly, three further certified translators each translated one Dutch translation back into US English. The resulting US English versions were compared to the originals and all discrepancies were discussed by three researchers (SAR, MRC and AGCV) who spoke both Dutch and English. Discrepancies were also discussed with the developers of the PSC, Dr J.M. Murphy and Dr M.S. Jellinek, especially where items raised questions as to their intended meaning. The PSC consists of 35 items that are rated as never, sometimes or often present (0, 1 and 2, respectively). Item scores are summed; we dichotomised at 0–27 vs. 28–70, following the US cut-off [10].

The CBCL was used to assess parents' reports of the behavioural and emotional problems of their children over the preceding six months. Its (good) reliability and validity has been established [18,19]. We used only the 120 problem items from the CBCL and computed scores for two broad-band groups of syndromes designated as Internalising and Externalising, and a Total Problems score. Children were also allocated to a normal range or a clinical range, using the 90th percentile of the Dutch normative sample as the cut-off [19].

### Analysis

In the analysis we assessed the psychometric properties of the PSC and its added value in identifying psychosocial problems. Regarding psychometric properties, we first computed its internal consistency and examined the fit between the scale structure and the observed data using confirmatory factor analysis (CFA) with structural equation modelling. Next, we assessed the validity of the PSC using dichotomised CBCL scores (Total Problems score and Internalising/Externalising scales) and referral by the CHP due to psychosocial problems as criteria. Finally, we assessed whether mean PSC scores differed with the children's background.

Regarding the added value of the PSC in identifying psychosocial problems, we assessed the odds of identification of mental health problems (i.e. a clinical CBCL Total Problems score) using an elevated score on the PSC. This was repeated with adjustment for social and demographic risk indicators known to the CHP that might have helped in the identification of psychosocial problems [3,4]. Regarding social and demographic risk indicators, we retained children with missing data in the logistic regression models by creating separate dummies for the missing category of each variable.

All analyses were done with SPSS 12.0 for Windows [21], except the CFA, which was done with Amos 5 [22]. All analyses were repeated for boys and girls separately. Results for these subgroups are provided only if they differed in a statistically significant way (p < 0.05).

## Results

### Demographics

The average age of the children in the study was 9.7 years (standard deviation 1.4 years) and there were slightly more girls than boys. Further demographic information is presented in Table 1.

### Scores on PSC and CBCL

Mean scores on the PSC are slightly higher for boys than for girls, which also holds for the CBCL (Table 2a). The internal consistency of the PSC was very good (Cronbach's alpha 0.89), though the CFA revealed that the items could not be fully represented by a single factor (Chi-square = 2715 at 560 df; p < 0.001; Goodness-of-Fit Index (GFI) = 0.75; Parsimony corrected GFI = 0.66).

Table 2b shows the prevalence rates of elevated scores on the same questionnaires using their established cut-offs [10,19]. Of all the children, 4.5% had elevated scores on the PSC and 8.9% had elevated scores on the CBCL. The latter closely resembles its distribution in the Dutch normative sample. In US populations, the prevalence of elevated PSC scores ranges from 12–14%. This corresponds to a cut-off of 0–21 vs. 22+ among Dutch children, when compared in Table 2b. To enable comparisons with US data on the PSC, all further analyses are presented for this cut-off too.

### Validity

**Figure 1:**
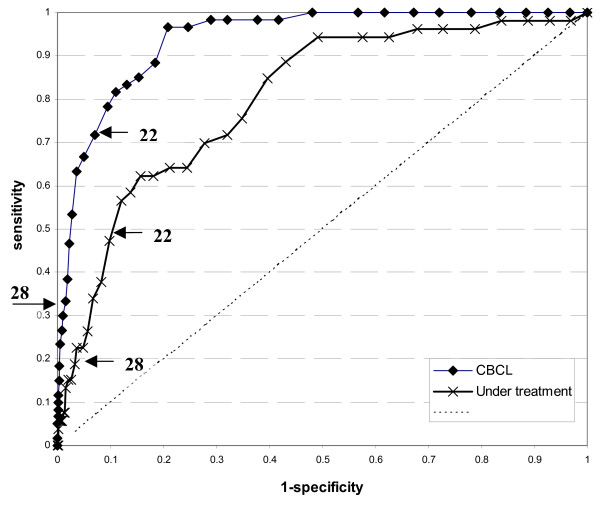
Receiver Operating Characteristic (ROC) curve for all possible cut-off points of the Dutch version of the PSC, using a clinical CBCL score and Currently Under Treatment or Psychosocial Problems as criteria.

Subsequently, the degree to which the score on the PSC is truly elevated in the case of psychosocial problems as measured by these four criteria (i.e. sensitivity) and the degree to which it is 'normal' in the case of the absence of these problems (i.e. specificity) were assessed. For the recommended cut-off of the PSC at 28 and above, scores were 0.33 and 0.98 respectively, using a clinical CBCL Total Problems score as the criterion, and 0.19 and 0.97 respectively, using being under treatment for mental health problems as the criterion. Figure 1 shows the Receiver Operating Characteristic (ROC) curve for all possible cut-off points. The curve is close to the upper-left corner of the figure, particularly when the CBCL is used as the criterion, indicating a high validity of the PSC if this gold standard is used. Curves for CBCL Internalising and Externalising Problems are largely similar but slightly more off the upper-left corner (i.e. less favourable; not shown). The same holds for problems detected by the CHP when compared with the curve for 'under treatment' (not shown). Table 3 shows the resulting areas under the ROC curves (AUC) and positive and negative predictive values for both cut-offs. Results regarding AUCs did not differ by gender (not shown).

### Differences in scores by background characteristics

Mean PSC scores were higher for boys, for children from minority backgrounds, single-parent families and unemployed families (Table 1, final columns).

### Added value

Finally, we examined the degree to which information from the PSC contributed to the diagnosis of psychosocial problems as measured by the CBCL over and above the clinical opinion of the child health physician without knowledge of the PSC. This yielded an odds ratio of 21.3 (95% confidence interval 8.7 to 52.2), with the only predictive background characteristic being family composition. Using the alternative PSC cut-off of 22+ yielded slightly higher odds ratios.

### Parent opinion of the PSC

A large majority of parents completed the PSC fully (91.1%) and no parent missed more than 3 items. However, 20% of parents made critical remarks about the PSC, mainly concerning lack of fit between questions and answer categories (7%) and unclear questions (5%).

## Discussion and conclusion

This study assessed the psychometric qualities of the Dutch version of the PSC and whether it is suitable for and contributes to the early detection of psychosocial problems among Dutch children aged 7–12 by PCH. Results reveal a good internal consistency and validity using the CBCL as gold standard. However, lower cut-offs have to be used for Dutch children than for children from the USA because of the Dutch children's on-average lower scores.

### Limitations

Methodological factors are unlikely to have affected these results. In general, the response rate was high (84%). Moreover, we used the CBCL as a criterion, which has been proven to be a valid measure for psychosocial problems. Because of complexity and high costs, structured clinical interviews such as the Diagnostic Interview Schedule for Children were not used as criteria [23]. Doing so may have provided additional information but differences with questionnaire-based information have been shown to be small [24].

### Fit with previous research on PSC and on other questionnaires used in PCH

This first study of the Dutch version of the PSC yielded results on reliability and on validity regarding the CBCL that are very similar to those found in comparable US samples. Jellinek et al. reported a sensitivity of 51.5% and a specificity of 95.4% at a cut-off of 0–27/28+, using the CBCL as a criterion in a sample of 206 children from the USA [9,25]. These values are very similar to those for a cut-off of 0–24/25+ for the Dutch version, i.e. 53.3% and 97.3% respectively (compare figure). We found the internal consistency of the PSC similar to that found by Jellinek et al. [25] but the results of our confirmatory factor analyses cast some doubt as to whether it measures a single latent, as did a previous study of Gardner et al. [26].

We found much lower mean scores on the PSC than have been found for comparable US samples. Mean scores on other symptom checklists such as the CBCL are also lower for Dutch children than for children from the USA [27]. Therefore, the Dutch children's lower mean PSC scores probably reflect real differences between these countries in the levels of symptoms reported by parents. This also implies that the cut-off for an elevated score on the PSC should be set lower for Dutch children than for children from the US. At this lower cut-off, the sensitivity and specificity of the Dutch version is similar to that of the US version. Moreover, the test characteristics of the PSC are comparable with or slightly better than those of most questionnaires currently used in Dutch PCH [15,16,28].

Finally, we found higher mean PSC scores for boys and for children from single-parent families, similar to those found by Jellinek et al. for children in the USA [10]. We did not find differences in terms of parental education level, in contrast to the findings of Jellinek et al. [10], but we did find elevated scores among children with unemployed parents, an indicator of familial socioeconomic status that was not studied by Jellinek et al. [10].

### Implications

The results of our study imply that the PSC is useful for the early detection of psychosocial problems by PCH, especially if an adjusted cut-off is used. The PSC mostly detects behavioural and emotional problems, which are common in this age group. However, questions on more extreme behaviours such as the abuse of alcohol and drugs are not asked. Screening using the PSC is best carried out as a first step in a two-step process on the way to referral. A relatively low-cut-off can then be used to avoid missing too many cases. In a second step, cases flagged by the PSC should then be assessed by a CHP before making a final decision about referral. Parental responses show that some questions may require revision. In any event, the PSC is a useful aid for the early detection of psychosocial problems that could be considered for use in other countries as well.

## Competing interests

The author(s) declare that they have no competing interests.

## Authors' contributions

SAR had the original idea for the project and wrote the study protocol. AGCV coordinated the study. All authors discussed the protocol and formulated the final design. MRC supervised the data collection. AGCV, FH, and SAR did the statistical analyses, which were discussed by all authors. SAR wrote the final manuscript, which was discussed, edited and revised by all authors. All authors read and approved the final manuscript.

## Appendix US version of the Pediatric Symptom checklist (Dutch version available on request from the authors)

Pediatric Symptom Checklist (PSC)

Emotional and physical health go together in children. Because parents are often the first to notice a problem with their child's behavior, emotions or learning, you may help your child get the best care possible by answering these questions. Please indicate which statement best describes your child.

Please mark under the heading that best describes your child:

NEVER SOMETIMES OFTEN

1. Complains of aches and pains..................................... 1 _______ _______ _______

2. Spends more time alone............................................. 2 _______ _______ _______

3. Tires easily, has little energy....................................... 3 _______ _______ _______

4. Fidgety, unable to sit still........................................... 4 _______ _______ _______

5. Has trouble with teacher........................................... 5 _______ _______ _______

6. Less interested in school........................................... 6 _______ _______ _______

7. Acts as if driven by a motor........................................ 7 _______ _______ _______

8. Daydreams too much................................................ 8 _______ _______ _______

9. Distracted easily....................................................... 9 _______ _______ _______

10. Is afraid of new situations........................................... 10 _______ _______ _______

11. Feels sad, unhappy................................................... 11 _______ _______ _______

12. Is irritable, angry..................................................... 12 _______ _______ _______

13. Feels hopeless......................................................... 13 _______ _______ _______

14. Has trouble concentrating......................................... 14 _______ _______ _______

15. Less interested in friends........................................... 15 _______ _______ _______

16. Fights with other children.......................................... 16 _______ _______ _______

17. Absent from school........................................... 17 _______ _______ _______

18. School grades dropping............................................ 18 _______ _______ _______

19. Is down on him or herself........................................... 19 _______ _______ _______

20. Visits the doctor with doctor finding nothing wrong..... 20 _______ _______ _______

21. Has trouble sleeping................................................. 21 _______ _______ _______

22. Worries a lot............................................................ 22 _______ _______ _______

23. Wants to be with you more than before........................ 23 _______ _______ _______

24. Feels he or she is bad................................................. 24 _______ _______ _______

25. Takes unnecessary risks............................................. 25 _______ _______ _______

26. Gets hurt frequently.................................................. 26 _______ _______ _______

27. Seems to be having less fun....................................... 27 _______ _______ _______

28. Acts younger than children his or her age..................... 28 _______ _______ _______

29. Does not listen to rules............................................. 29 _______ _______ _______

30. Does not show feelings............................................. 30 _______ _______ _______

31. Does not understand other people's feelings................. 31 _______ _______ _______

32. Teases others.......................................................... 32 _______ _______ _______

33. Blames others for his or her troubles........................... 33 _______ _______ _______

34. Takes things that do not belong to him or her.............. 34 _______ _______ _______

35. Refuses to share...................................................... 35 _______ _______ _______

Total score ______________

Does your child have any emotional or behavioral problems for which she/he needs help? () N () Y

Are there any services that you would like your child to receive for these problems? () N () Y

If yes, what services? _________________________________________________________

## Pre-publication history

The pre-publication history for this paper can be accessed here:



## Figures and Tables

**Table 1:** Demographic characteristics of the participating children, and mean Pediatric Symptom Checklist scores for selected socio-demographic groups (n = 674)*.

**Characteristic**	No. (%) *	**Mean**	**SD**	P-value **
Age				
Gender				
Male	327 (48.2%)	12.5	8.4	0.005
Female	347 (51.8%)	10.8	7.7	
Ethnic background				
Dutch	553 (91.3%)	11.3	7.8	0.034
Immigrant/minority	53 (8.7%)	13.7	8.5	
Family composition				<0.0001
Two parents	558 (85.5%)	10.9	7.7	
Single parent	78 (11.9%)	15.7	8.4	
Other	17 (2.6%)	13.8	7.1	
Highest parental education				0.57
Only primary school (=8 years)	21 (3.3%)	11.9	5.7	
Lower vocational (=max. 12 years)	182 (28.6%)	11.3	7.7	
Higher vocational (=max. 16 years)	196 (30.8%)	11.0	7.3	
University/higher professional (17 years and over)	238 (37.4%)	12.0	8.8	
Parental employment status				0.016
No paid employment	38 (5.6%)	15.1	11.7	
One parent with paid employment	193 (28.6%)	11.8	7.8	
Two parents with paid employment	443 (65.7%)	11.2	7.7	

* Numbers do not always total 674 because of missing values.

** P-value for differences in mean scores by background characteristic.

**Table 2a:** Scores on the PSC and CBCL Total Problems, Internalising and Externalising scales, for all children and by gender (mean, standard deviation, range).

**Scale**	**Total (n = 674)**			**Boys (n = 327)**			**Girls (n = 347)**		
	mean	(SD)	range	mean	(SD)	Range	mean	(SD)	range
PSC	11.6	(8.1)	0–46	12.5	(8.4)	0–40	10.8	(7.7)	0–46
CBCL									
Total	18.7	(15.3)	1–118	20.1	(16.7)	1–118	17.3	(13.9)	1–74
Internalising	5.4	(5.5)	0–35	5.3	(5.5)	0–35	5.6	(5.5)	0–31
Externalising	5.7	(6.0)	0–34	6.7	(6.8)	0–34	4.8	(5.0)	0–25

**Table 2b:** Numbers and percentages of children with elevated scores on the PSC and CBCL Total Problems, Internalising and Externalising scales, and of children currently under treatment for psychosocial problems, for all children and by gender.

**Scale**	**Total (n = 674)**	**Boys (n = 327)**	**Girls (n = 347)**
PSC^a^	30 (4.5%)	18 (5.5%)	12 (3.5%)
PSC adjusted^b^	86 (12.8%)	53 (16.2%)	33 (9.5%)
CBCL			
Total^c^	60 (8.9%)	32 (9.8%)	28 (8.1%)
Internalising^d^	82 (12.2%)	40 (12.2%)	42 (12.1%)
Externalising^e^	52 (7.7%)	28 (8.9%)	23 (6.6%)
Currently under treatment for psychosocial problem	53 (7.9%)	34 (10.4%)	19 (5.5%)

^a ^28 and over for boys and girls (i.e. original US cut-off)

^b ^22 and over for boys and girls (i.e. adjusted Dutch cut-off)

^c ^38 and over for boys and girls

^d ^13 and over for boys, and 16 and over for girls

^e ^14 and over for boys, and 13 and over for girls

All cut-offs refer to the age groups studied.

**Table 3:** Sensitivity (Sens.), specificity (spec.), AUC and positive and negative predictive value (PPV, NPV) for the PSC at cut-off 27, using CBCL Total, Internalising and Externalising Problems, and under treatment for psychosocial problems as criteria (n = 674).

	**Cut-off 0–27 vs. 28+**				**Cut-off 0–21 vs. 22+**				**AUC**	**(95% CI)**
	Sens.	Spec.	PPV	NPV	Sens.	Spec.	PPV	NPV		
CBCL										
Total^b^	33.3%	98.4%	66.7%	93.8%	71.7%	93.0%	50.0%	97.1%	0.94	(0.92–0.96)
Internalising^c^	24.4%	98.3%	66.7%	90.4%	53.7%	92.9%	51.2%	93.5%	0.89	(0.86–0.92)
Externalising^d^	30.8%	97.7%	53.3%	94.4%	57.7%	91.0%	34.9%	96.3%	0.90	(0.87–0.93)
Currently under treatment	18.9%	96.8%	33.3%	93.3%	47.2%	90.2%	29.1%	95.2%	0.80	(0.74–0.86)
